# Pre-pregnancy BMI modifies the optimal interpregnancy interval for preventing preterm birth: a population-based retrospective cohort study

**DOI:** 10.3389/fendo.2026.1762209

**Published:** 2026-06-12

**Authors:** Min-Yi Yuan, Si-Liang Zeng, Wei-Chao He

**Affiliations:** Dongguan Maternal and Child Health Care Hospital, Dongguan, Guangdong, China

**Keywords:** body mass index, cohort study, interpregnancy interval, pre-pregnancy, preterm birth

## Abstract

**Objective:**

To investigate whether pre-pregnancy body mass index (BMI) modifies the association between interpregnancy interval (IPI) and preterm birth (PTB) risk and to determine the BMI-specific IPI associated with the lowest PTB risk.

**Methods:**

This population-based retrospective cohort study utilized data from 127,542 multiparous women in Dongguan, China (2014-2024). We employed two complementary approaches: a generalized additive mixed model and generalized estimating equations with restricted cubic splines, to examine the interaction between IPI and pre-pregnancy BMI on PTB. The robustness of the findings was evaluated through inverse probability weighting and subgroup analyses.

**Results:**

Pre-pregnancy BMI significantly modified the IPI-PTB association. A clear inverse relationship was observed, whereby the IPI associated with the lowest PTB risk shortened progressively as BMI increased. The adjusted IPI associated with the lowest PTB risk decreased from 38.8 months (95% CI: 32.0-60.0) among underweight women to 32.4 months (29.7-37.2) for normal weight and 26.7 months (6.0-30.8) for overweight. For women with obesity, within the studied range (IPI ≥ 6 months), the lowest risk was observed at the shortest interval (6.0 months), although the wide 95% confidence interval (6.0-60.0) and the exclusion of IPIs **<**6 months warrant cautious interpretation. This **“**dose-response**”** pattern remained robust across sensitivity and subgroup analyses.

**Conclusion:**

This study provides evidence that pre-pregnancy BMI is an important effect modifier of the IPI-PTB relationship. The finding that the IPI associated with the lowest PTB risk shortens with increasing BMI indicates that the relationship between interpregnancy interval and preterm birth varies by maternal BMI. Although causal inference cannot be drawn from this observational study, the results suggest that uniform IPI guidelines may warrant re-examination across BMI categories.

## Introduction

Preterm birth (PTB) is a major adverse perinatal outcome (1), and the precision of its prevention strategies is crucial for achieving optimal maternal and child health. The interpregnancy interval (IPI), as an important modifiable factor, has been widely demonstrated to exhibit a U-shaped relationship with the risk of PTB (2–4), giving rise to the concept of an “optimal interval” However, current clinical recommendations are primarily based on population averages, overlooking significant interindividual heterogeneity. Emerging evidence suggests that this heterogeneity partly stems from the potential modifying effects of maternal baseline characteristics on the IPI-PTB relationship (2, 3).

Against this backdrop, the role of pre-pregnancy body mass index (BMI), a core and quantifiable indicator of maternal physiological status (5), remains unclear. BMI is not only independently associated with the risk of PTB (6) but may also fundamentally influence a mother’s physiological readiness for a subsequent pregnancy by affecting metabolic diseases (7), and intrauterine environmental homeostasis (8). To date, no studies have systematically investigated whether and how pre-pregnancy BMI modifies the association between IPI and PTB risk, nor have they addressed the key clinical question of the BMI-specific IPI associated with the lowest PTB risk for women.

Therefore, this study investigates pre-pregnancy BMI as a key effect modifier of the IPI-PTB association. Using a large-scale cohort dataset from Dongguan and advanced statistical models, we aim to achieve three core objectives: (1) rigorously examine the interaction between IPI and pre-pregnancy BMI on PTB risk; (2) precisely delineate the dynamic trajectory of the IPI associated with the lowest PTB risk across the full spectrum of pre-pregnancy BMI; and (3) systematically evaluate the robustness of this association across key clinical subgroups.

## Materials and methods

### Data source and study design

This retrospective cohort study utilized data from the Dongguan Maternal and Child Health Information Platform, covering the period from January 1, 2014, to December 31, 2024. The platform encompasses all medical institutions providing obstetric services in Dongguan, ensuring a city-wide representative population.

For the purpose of longitudinal matching of IPIs, the initial dataset was structured into two sub-cohorts: Cohort A (Historical Matching Pool): Comprised delivery records from 2014 to 2018. This cohort was used solely as a pool for calculating the IPI due to the systematic absence of BMI data and was not included in the final risk analysis. Cohort B (Core Analysis Cohort): Comprised delivery records from 2019 to 2024. This cohort served as the starting point for matching and constituted the core population for the final risk analysis.

Study participants were defined as multiparous women with at least two consecutive delivery records during the specified period, identified through longitudinal matching based on maternal national ID numbers.

### Definitions

① IPI: Defined as the time interval (in months) from the preceding delivery date to the conception date of the index pregnancy. It was calculated using the formula: IPI (months) = [Date of index delivery-Date of prior delivery-Gestational age (in days) of the index pregnancy]/30.44. ② BMI: In this study, pre-pregnancy weight was estimated as follows. For the majority of women whose first antenatal visit occurred within the first trimester (≤12 weeks), because weight change from pre-pregnancy is minimal, the measured weight at that visit was used as an approximation. For the few women with first visits after the first trimester, recalled pre-pregnancy weight was used instead. BMI was calculated as weight (kg)/[height (m)]². For subgroup analysis, pre-pregnancy BMI was categorized as follows: underweight (16.0-18.4 kg/m²), normal weight (18.5-23.9 kg/m²), overweight (24.0-27.9 kg/m²), and obese (28.0-32.0 kg/m²). ③ PTB: Defined as a delivery occurring at or after 28 weeks and before 37 completed weeks of gestation (i.e., from 196 days to 258 days) of the index pregnancy, in accordance with China’s perinatal surveillance standard. ④ Gestational age: Gestational age was determined by first-trimester ultrasound (crown-rump length) in the majority of cases; when ultrasound was not available, it was based on last menstrual period confirmed by early ultrasound.

### Inclusion and exclusion criteria

Inclusion Criteria: Multiparous women with two consecutive delivery records between 2014 and 2024 in the Dongguan Maternal and Child Health Information Platform, who were successfully matched based on national ID numbers.

Exclusion Criteria (applied sequentially): ① Individuals with missing height or weight data, or with recorded values deemed physiologically implausible. ② Individuals with an IPI outside the predefined analytical range for this study (i.e., < 6 months or > 60 months). IPIs <6 months were excluded because such intervals are associated with increased risks of uterine rupture (especially after cesarean delivery) and maternal nutritional depletion, making them clinically contraindicated rather than merely suboptimal. ③ Individuals with extreme BMI values (i.e., < 16 or > 32) calculated at the first antenatal visit of the index pregnancy.

### Data collection and management

Successive pregnancies were linked through matching maternal national identifiers to establish longitudinal records. To ensure data consistency, this study utilized data from two sources: System 3 (2019-2024) served as the primary data source providing BMI measurements and other key variables, while System 2 (2014-2018) was used exclusively for IPI calculation through record linkage. The integrated dataset encompassed maternal demographics (age, residence, partner’s age), clinical history (previous pregnancy loss, PTB, and cesarean delivery), current pregnancy characteristics (twin gestation, pre-pregnancy BMI, use of assisted reproductive technology (ART)), and delivery outcomes (date of delivery, gestational age). Rigorous quality control measures were implemented throughout the process, with two researchers independently performing data extraction and consolidation. Discrepancies were resolved through arbitration by a senior investigator, followed by systematic screening to identify and address outliers and physiologically implausible values.

### Statistical analysis

All statistical analyses were performed using R (version 4.5.0). Descriptive statistics summarized baseline characteristics. Normality of continuous variables was assessed using the Shapiro-Wilk test. Variables following a normal distribution were reported as mean (SD), while non-normally distributed variables were reported as median (interquartile range, IQR). Categorical variables were reported as numbers and percentages. Crude PTB rates were visualized via a heatmap stratified by pre-pregnancy BMI categories and IPI groups, presented as percentages with sample sizes. For comparisons of baseline characteristics between groups, continuous variables were first assessed for normality using the Shapiro-Wilk test. Normally distributed variables were compared using one-way ANOVA (with Levene’s test for homogeneity of variances); non-normally distributed variables were compared using the Kruskal-Wallis H test. Categorical variables were compared using the chi-square test or Fisher’s exact test (when expected cell counts <5). All hypothesis tests were two-sided with a significance level of α=0.05.

The association between IPI, maternal BMI, and PTB was analyzed using two complementary approaches. First, a generalized additive mixed model (GAMM) (9) was applied, incorporating a tensor product smooth term for IPI and BMI, and adjusted for maternal age, paternal age, residence, twin pregnancy, obstetric history (PTB, cesarean delivery, abortion), and ART. Model selection was based on the Akaike Information Criterion (AIC). An unadjusted GAMM including only the IPI-BMI smooth term was also fitted for comparison. Second, generalized estimating equations (GEE) with restricted cubic splines (RCS) were used to examine the non-linear relationship between IPI and PTB across BMI categories (10). The number of knots for RCS was determined using the Quasi-likelihood under the Independence model Criterion (QIC). The fully adjusted model controlled for residence, paternal age, maternal age, history of abortion, twin pregnancy, history of PTB, and ART, while an unadjusted version was fitted for comparative purposes.

To assess robustness, inverse probability weighting (IPW) (11) sensitivity analyses were conducted for both GAMM and GEE+RCS models. Propensity scores were estimated per BMI group via multinomial logistic regression including the aforementioned covariates. We computed stable inverse probability weights, trimmed at the 1st and 99th percentiles, and incorporated into weighted models. Consistency between primary and IPW-derived IPIs associated with the lowest PTB risk was evaluated using bootstrap 95% confidence intervals for the difference, with clinical equivalence predefined as within ±3 months. Subgroup analyses were performed using the GEE+RCS framework in four clinically relevant populations: advanced maternal age (≥35 years), and women with a history of abortion, cesarean section, or PTB. These models were similarly adjusted for covariates, with relevant historical factors omitted from the adjustment set when they defined the subgroup itself. A *post-hoc* simulation-based power analysis was performed for the joint interaction effect (IPI × BMI category) using the fitted GEE model. Missing data were handled throughout using multiple imputation with five imputed datasets using predictive mean matching (PMM) and 10 iterations.

## Results

### Study population characteristics

Following the longitudinal matching process, 179,158 multiparous women were successfully identified. After applying the predefined exclusion criteria, 10,269 women were excluded due to missing or implausible height/weight data, and a further 38,993 were excluded for having an IPI outside the study range (IPI >60 months, n=34,541; IPI <6 months, n=4,452). Finally, 2,354 women with extreme BMI values (BMI >32, n=1,138; BMI <16, n=1,216) were excluded. Consequently, the final study population for analysis comprised 127,542 women. Baseline characteristics of the study population are shown in **Table 1**. **Supplementary Material 1** illustrates the participant selection process. The distribution of crude PTB rates across categories of IPI and maternal pre-pregnancy BMI is presented in **Supplementary Material 2**. The data suggest a pattern where the IPI associated with the lowest preterm rate shortens with increasing BMI. For underweight women (BMI 16.0-18.4), the lowest rates (4.7%-4.9%) were observed at intermediate to long IPIs (18-47.9 months). In contrast, for women with obesity (BMI 28.0-32.0), the lowest rate (6.6%) occurred at a much shorter IPI (12-17.9 months), with a pronounced increase to 8.5% at the longest interval (48–60 months).

**Table 1:** Baseline characteristics of the study population (N = 127,542).

Variable	Total	Group1 (16–<18.5)	Group2 (18.5–<24)	Group3 (24–<28)	Group4 (28–32)	Statistic	P
Maternal age (years)						H = 2374.02	<0.001
Mean ± SD	29.72 ± 4.32	28.50 ± 4.01	29.81 ± 4.27	30.45 ± 4.50	30.43 ± 4.55		
Median (IQR)	30.00 (27.00–32.00)	28.00 (26.00–31.00)	30.00 (27.00–33.00)	30.00 (27.00–33.00)	30.00 (27.00–33.00)		
Paternal age (years)						H = 1989.90	<0.001
Mean ± SD	31.95 ± 4.86	30.76 ± 4.53	32.01 ± 4.84	32.68 ± 4.98	32.99 ± 5.09		
Median (IQR)	32.00 (29.00–35.00)	30.00 (28.00–33.00)	32.00 (29.00–35.00)	32.00 (29.00–35.00)	32.00 (29.00–36.00)		
IPI (months)						H = 278.22	<0.001
Mean ± SD	29.84 ± 14.75	28.31 ± 14.53	30.19 ± 14.75	29.96 ± 14.85	30.00 ± 14.85		
Median (IQR)	28.20 (17.10–41.70)	25.90 (15.80–39.50)	28.70 (17.60–42.00)	28.30 (17.10–41.90)	28.10 (17.30–41.80)		
BMI (kg/m²)						H = 93825.59	<0.001
Mean ± SD	21.47 ± 3.08	17.53 ± 0.67	20.96 ± 1.49	25.48 ± 1.11	29.48 ± 1.12		
Median (IQR)	21.00 (19.20–23.30)	17.60 (17.10–18.10)	20.80 (19.70–22.20)	25.30 (24.50–26.40)	29.30 (28.50–30.30)		
Preterm birth						χ² = 102.71	<0.001
Yes	6689 (5.2%)	1048 (5.0%)	3981 (4.9%)	1307 (6.2%)	353 (7.4%)		
No	120853 (94.8%)	19745 (95.0%)	76961 (95.1%)	19709 (93.8%)	4438 (92.6%)		
Urban residence						χ² = 39.48	<0.001
Urban	52007 (40.8%)	8629 (41.5%)	33293 (41.1%)	8201 (39.0%)	1884 (39.3%)		
Rural	75427 (59.2%)	12142 (58.4%)	47589 (58.8%)	12793 (60.9%)	2903 (60.6%)		
Multiple gestation						χ² = 28.63	<0.001
Yes	1253 (1.0%)	145 (0.7%)	805 (1.0%)	254 (1.2%)	49 (1.0%)		
No	126289 (99.0%)	20648 (99.3%)	80137 (99.0%)	20762 (98.8%)	4742 (99.0%)		
Prior preterm birth						χ² = 43.79	<0.001
Yes	6287 (4.9%)	1053 (5.1%)	3797 (4.7%)	1126 (5.4%)	311 (6.5%)		
No	121255 (95.1%)	19740 (94.9%)	77145 (95.3%)	19890 (94.6%)	4480 (93.5%)		
Prior cesarean delivery						χ² = 1966.46	<0.001
Yes	34476 (27.0%)	3893 (18.7%)	21226 (26.2%)	7345 (34.9%)	2012 (42.0%)		
No	93066 (73.0%)	16900 (81.3%)	59716 (73.8%)	13671 (65.1%)	2779 (58.0%)		
History of abortion						χ² = 188.16	<0.001
Yes	47577 (37.3%)	6986 (33.6%)	30346 (37.5%)	8314 (39.6%)	1931 (40.3%)		
No	79938 (62.7%)	13805 (66.4%)	50581 (62.5%)	12694 (60.4%)	2858 (59.7%)		
ART						χ² = 102.37	<0.001
Yes	1436 (1.1%)	115 (0.6%)	922 (1.1%)	324 (1.5%)	75 (1.6%)		
No	126106 (98.9%)	20678 (99.4%)	80020 (98.9%)	20692 (98.5%)	4716 (98.4%)		

Continuous variables are presented as mean ± SD and median (IQR) because all were non-normally distributed (Shapiro–Wilk test P < 0.001). H: Kruskal-Wallis chi-square statistic; χ²: Pearson chi-square statistic. IPI, interpregnancy interval; ART, assisted reproductive technology. BMI groups, underweight (16–<18.5), normal (18.5–<24), overweight (24–<28), obese (28–32) kg/m². Data were missing for maternal age (0.1%), paternal age (1.0%), and history of abortion (0.02%). All missing data were handled using multiple imputation.

### GAMM analysis

**Figure 1:**
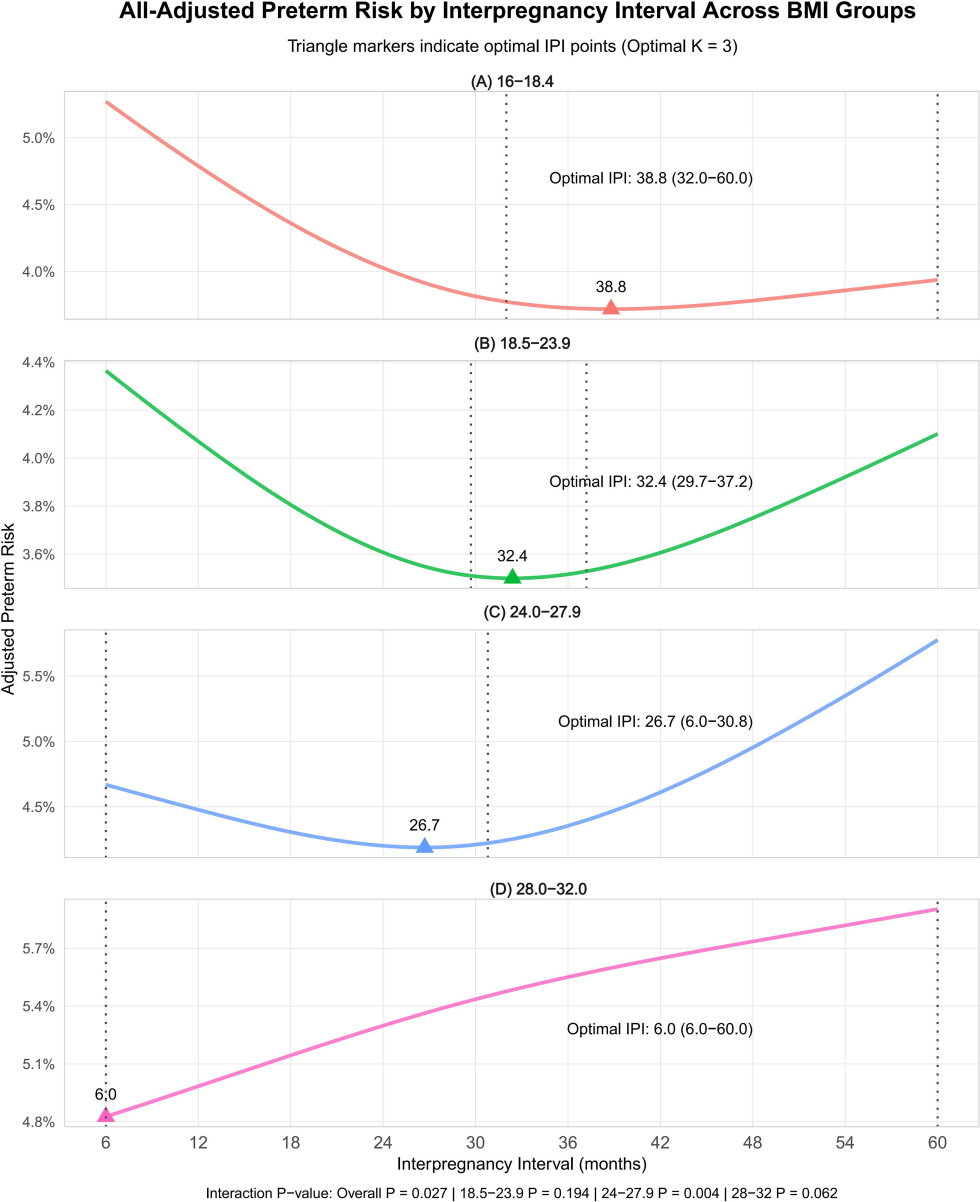
Joint Association of IPI and Maternal BMI with PTB Risk in the All-adjusted Model **(A)** Heatmap visualizing the adjusted association between IPI and maternal BMI with predicted PTB risk. The model is adjusted for maternal age, husband’s age, residence, twin pregnancy, obstetric history, and assisted reproductive technology. Warmer colors indicate higher risk. **(B)** Marginal effect of IPI on PTB Risk, stratified by specific maternal BMI levels. **(C)** Marginal effect of maternal BMI on PTB Risk, stratified by specific IPI lengths. **(D)** Optimal IPI (point of lowest predicted risk) as a function of maternal BMI, derived from the All-adjusted model, demonstrating a shortening of the optimal interval with increasing BMI.

The All-adjusted GAMM revealed a significant non-linear interaction between IPI and maternal BMI on PTB risk (**Figure 1**). The risk surface demonstrated that the relationship between IPI and PTB was modified by maternal BMI level. Marginal effect analyses showed that the risk profile differed across BMI strata. Crucially, the model-derived IPI associated with the lowest predicted risk exhibited a clear inverse relationship with maternal BMI. In this adjusted model, the IPI associated with the lowest predicted risk shortened progressively from approximately 45 months at a BMI of 16 to about 20 months at a BMI of 32, suggesting that women with a higher pre-pregnancy BMI may benefit from a shorter IPI to minimize PTB risk. The unadjusted model yielded results highly consistent with the All-adjusted analysis (**Supplementary Material 3**).

### GEE+RCS

**Figure 2:**
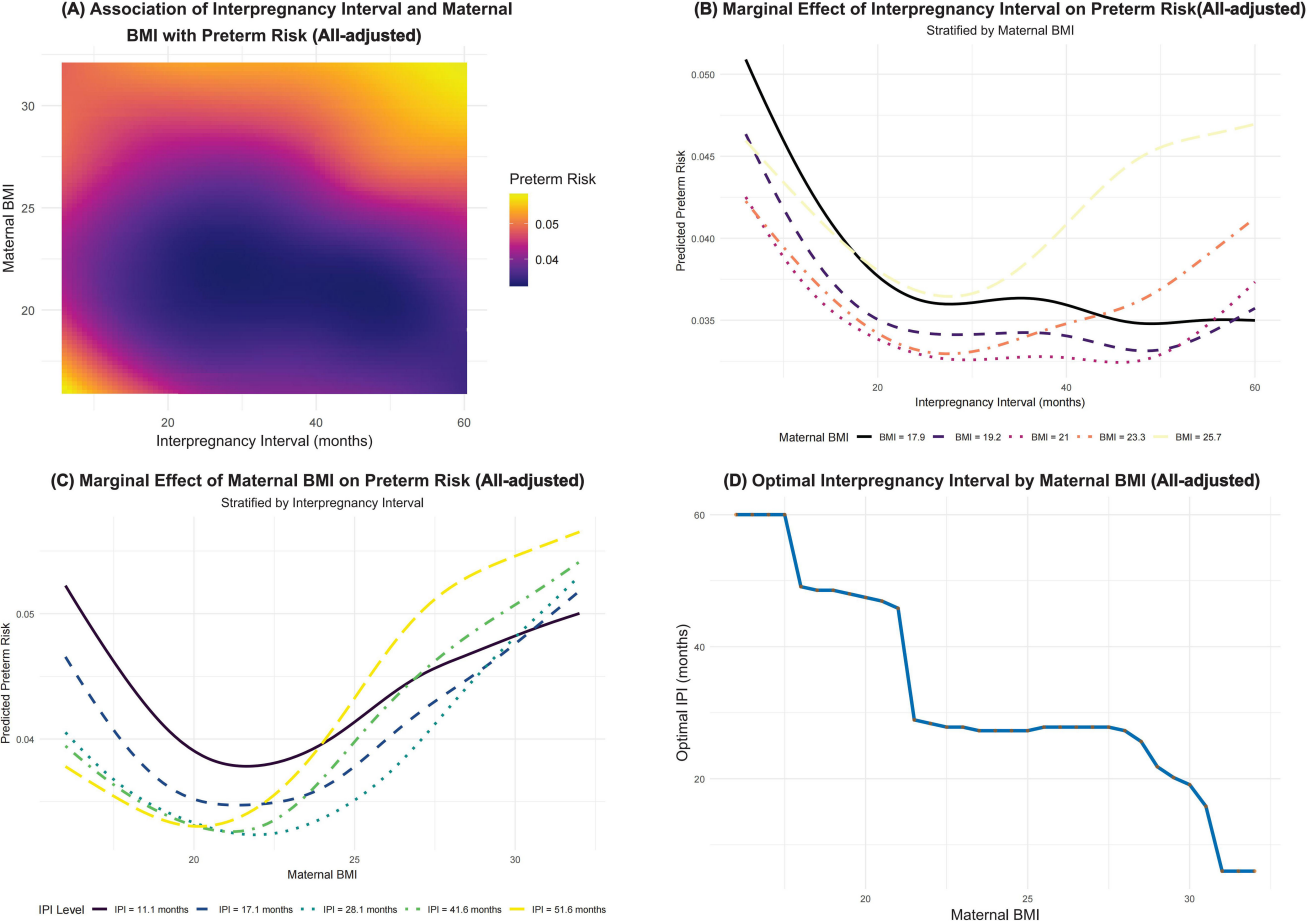
Adjusted PTB Risk by IPI, Stratified by Maternal BMI. The figure presents the results from the all-adjusted GEE model with RCS (optimal K = 3). **(A–D)** The curves depict the non-linear relationship between IPI and the predicted probability of PTB across four pre-pregnancy BMI categories. The model was adjusted for maternal age, paternal age, residence, history of abortion, twin pregnancy, history of PTB, and assisted reproductive technology. Triangle markers indicate the model-derived optimal IPI (point of lowest predicted risk) for each BMI group, with the numerical value and its 95% confidence interval (in parentheses) displayed above each panel. The dotted vertical lines represent the bounds of the 95% confidence interval for each optimal IPI.

The all-adjusted GEE model revealed a significant interaction between IPI and BMI categories on PTB risk (**Figure 2**). The IPI associated with the lowest PTB risk demonstrated a clear inverse relationship with increasing BMI: from 38.8 months (95% CI: 32.0-60.0) in women with BMI 16-18.4 to 32.4 months (29.7-37.2) in those with BMI 18.5-23.9, 26.7 months (6.0-30.8) in those with BMI 24.0-27.9, and only 6.0 months (6.0-60.0) in those with BMI 28.0-32.0. Of note, for the obese group, the relationship between IPI and PTB risk was monotonic increasing within the studied range, with the lowest risk observed at the shortest analyzable interval (6 months). However, because IPIs <6 months were excluded, this estimated optimum lies at the boundary of our data, and the wide confidence interval (6.0-60.0) reflects substantial uncertainty. Significant interactions were particularly observed for overweight women (P = 0.004), indicating substantially different IPI-PTB risk relationships across BMI strata. The unadjusted model showed consistent patterns, with the IPI associated with the lowest PTB risk similarly decreasing from 34.4 to 6.0 months across increasing BMI categories, but the same caution applies to the obese group estimate (**Supplementary Material 4**). *Post-hoc* power analysis for the IPI × BMI interaction effect demonstrated that the study had 92.6% power at α = 0.05, confirming adequate sample size for the primary analysis.

### IPW analyses

**Figure 3:**
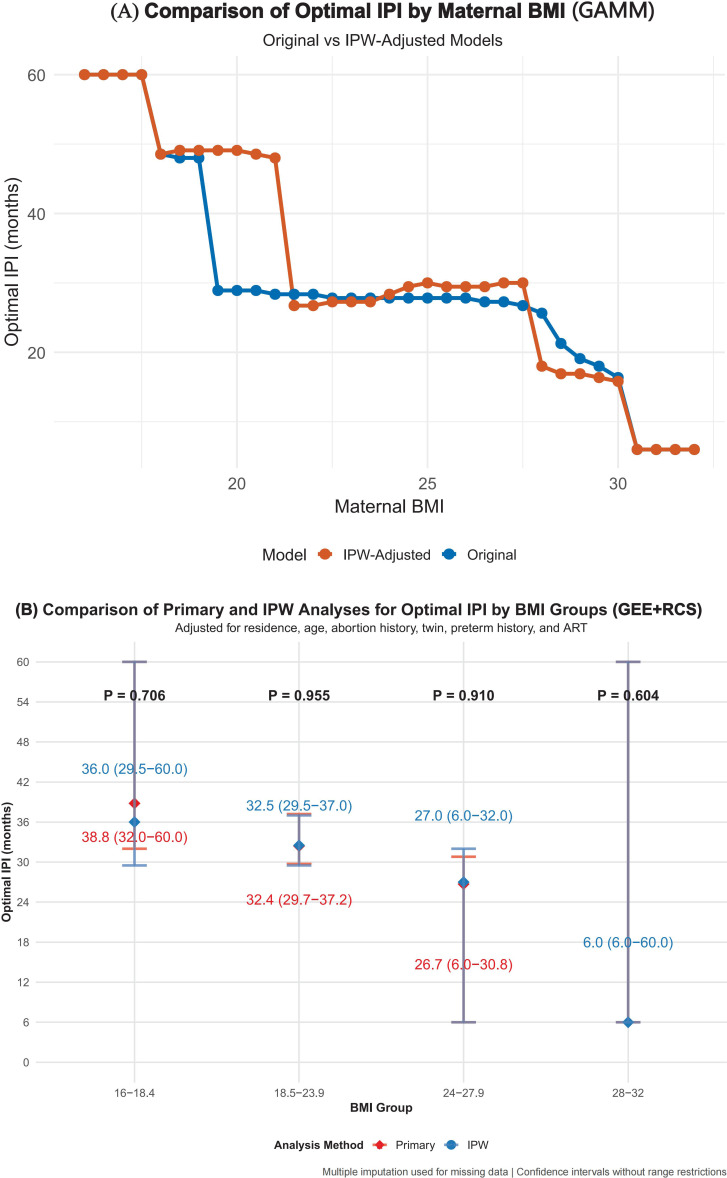
Sensitivity analysis using IPW. **(A)** Comparison of optimal IPI between original and IPW-adjusted GAMM models across the BMI continuum. **(B)** Comparison of optimal IPI (point estimate and 95% CI) between primary and IPW-adjusted GEE+RCS analyses, stratified by BMI category. P-values from consistency tests are shown above each group.

The IPW analyses yielded highly consistent results with the primary analyses for both modeling approaches (**Figure 3**). In the GAMM, the curve of the IPI associated with the lowest risk across the BMI continuum from the IPW-adjusted model closely aligned with that from the primary model. In the IPW analysis of GEE+RCS (adjusted for residence, age, abortion history, twin pregnancy, preterm history, and ART), no statistically significant differences were observed for any BMI group (all *P* > 0.05), confirming the robustness of the main findings.

### Subgroup analyses

**Figure 4:**
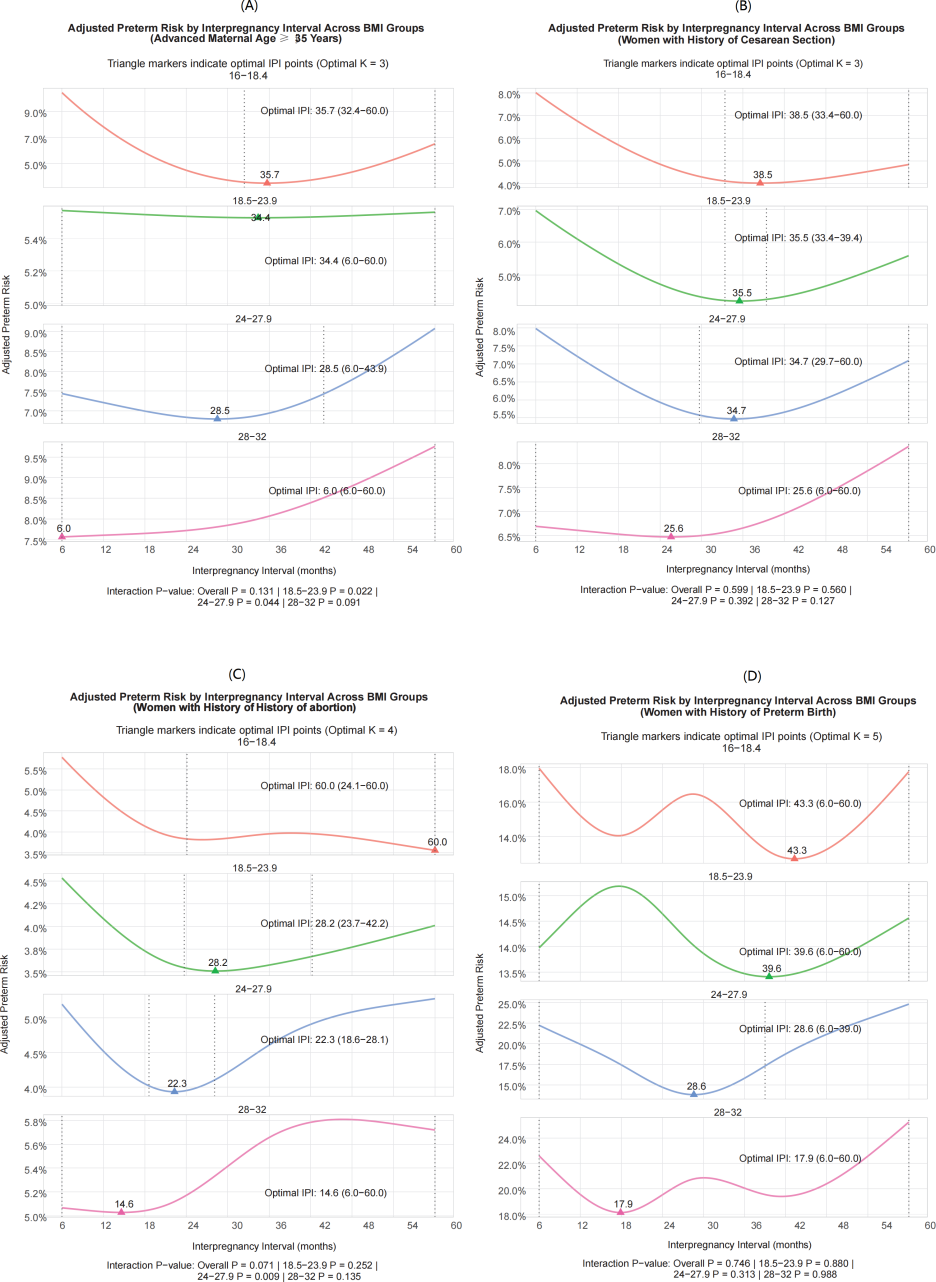
Subgroup analyses of optimal IPI by BMI Categories. **(A)** Advanced maternal age (≥35 years), **(B)** History of cesarean section, **(C)** History of abortion, **(D)** History of PTB. Each panel shows the adjusted PTB Risk curves across IPI for four BMI categories, with triangle markers indicating optimal IPI points. Numerical values represent optimal IPI (months) with 95% confidence intervals in parentheses. All models were adjusted for residence, age, abortion history (except panel B), twin pregnancy, preterm history [except **(D)**], and ART.

Stratified analyses consistently demonstrated an inverse relationship between maternal BMI and the IPI associated with the lowest PTB risk across all subgroups (**Figure 4**), aligning with the primary findings. Among advanced-age women (**Figure 4A**), the IPI associated with the lowest PTB risk shortened progressively from 35.7 months (95% CI: 32.4-60.0) in underweight women to 6.0 months (6.0-60.0) in obese women. In the cesarean section history subgroup (**Figure 4B**), the IPI associated with the lowest PTB risk ranged from 38.5 months (33.4-60.0) to 25.6 months (6.0-60.0). Similarly, for women with abortion history (**Figure 4C**), the IPI associated with the lowest PTB risk decreased from 60.0 months (24.1-60.0) to 14.6 months (6.0-60.0) across increasing BMI categories. Among women with previous PTB (**Figure 4D**), the IPIs associated with the lowest PTB risk varied from 43.3 months (6.0-60.0) to 17.9 months (6.0-60.0). All subgroups maintained the consistent pattern of shorter intervals associated with the lowest PTB risk with higher BMI levels.

## Discussion

### Summary of main findings

This large population-based study preliminarily investigated the potential modifying effect of pre-pregnancy BMI on the relationship between IPI and PTB risk. The core finding indicates that pre-pregnancy BMI appears to be a significant modifier of this association: the IPI associated with the lowest PTB risk demonstrated a clear shortening trend as maternal pre-pregnancy BMI increased. This “dose-response” relationship, consistently revealed by two complementary statistical modelling approaches, remained robust across multiple sensitivity analyses and key clinical subgroups. This suggests the potential importance of considering individual physiological characteristics when formulating IPI recommendations.

### Interpretation of findings

The core finding of this study is that pre-pregnancy BMI significantly modifies the association between IPI and PTB risk, demonstrating a progressive shortening of the IPI associated with the lowest PTB risk as BMI increases. This pattern suggests that women with low and high BMI may follow distinct postpartum physiological recovery trajectories. One hypothesis is that the shorter ideal IPI in women with obesity relates to their specific pathophysiological state, which may include chronic inflammation dysregulation. Potential manifestations could include significant postpartum weight retention (12, 13), progressive insulin resistance (14), elevated inflammatory markers such as CRP (15), increased risk of cardiometabolic diseases (7), and a sustained pro-inflammatory endometrial environment (16, 17). It is possible that this metabolic-inflammatory milieu persists well into the postpartum period (18). If so, prolonging the IPI might not facilitate recovery but could instead extend exposure to this adverse state, potentially leading to cumulative damage.

Conversely, women with a low pre-pregnancy BMI generally require a longer IPI to achieve comprehensive physiological recovery postpartum. This extended period is crucial for rebuilding nutrient reserves—not only for replenishing key nutrients such as folate (19) and ferritin (20) but also for restoring other micronutrients essential (21) for fetal development and placental function. Furthermore, underweight women often have limited metabolic reserves and relatively weaker recovery capacity (22), making them more susceptible to maternal nutrient depletion and health deterioration during lactation (23). For this population, a short IPI might be associated with increased maternal and fetal risks. A subsequent pregnancy initiated before full nutritional recovery may elevate the likelihood of fetal growth restriction and PTB. This line of reasoning would suggest that a longer IPI for underweight women might help ensure adequate physiological recuperation and could establish a more favorable intrauterine environment for the next pregnancy. However, these mechanistic interpretations are speculative and require confirmation in future studies.

### Comparison with literature

While some prior studies on optimal IPI have included BMI (24, 25), they treated it predominantly as a covariate for adjustment rather than as an effect modifier. Our findings align with the classical discourse on the U-shaped relationship between IPI and adverse pregnancy outcomes (2–4), but they further reveal the dynamic nature of this relationship as modified by maternal baseline BMI. Although previous studies have noted heterogeneity potentially introduced by maternal characteristics (26), this study is the first to systematically delineate the trajectory of the IPI associated with the lowest PTB risk across the entire BMI spectrum using large-scale data, thereby providing crucial evidence for ‘individualized IPI recommendations’ and addressing a significant gap in this field of research.

### Clinical and public health implications

The findings of this study offer insights for future research directions rather than immediate clinical changes. At the clinical level, our results do not support modifying current IPI recommendations, but they do suggest that further investigation—particularly prospective studies—is warranted to examine whether uniform IPI guidelines should be differentiated by maternal BMI. This implies that, instead of adhering to a uniform recommendation, the potential benefits of a shorter IPI (e.g., 12–18 months) might be explored for women with obesity, while a longer interval could be more suitable for underweight women. At the public health level, our data provide preliminary evidence to inform the future refinement of relevant guidelines, hinting at the potential of incorporating pre-pregnancy BMI into IPI recommendations. Furthermore, this study reinforces the importance of enhancing pre-conception care and weight counseling within primary healthcare systems (27), with the aim of helping more women plan pregnancies from a more favorable health baseline. However, for women with a prior cesarean delivery, a very short interpregnancy interval (≤6 months) increases the risk of uterine rupture.

### Strengths and limitations

This study has several notable strengths. First, the large-scale, population-based retrospective cohort design provided sufficient statistical power to detect the complex interaction between IPI and pre-pregnancy BMI. Second, the complementary use of both the GAMM and GEE+RCS allowed for a comprehensive characterization of the exposure-outcome relationship. The highly consistent findings from these two methods significantly enhanced the robustness of our results. Third, multiple sensitivity and subgroup analyses were conducted, further validating the reliability of the observed associations.

However, this study also has several limitations. The primary limitation stems from its retrospective observational design, which carries the potential for residual confounding. Second, the pre-pregnancy BMI was calculated using pre-pregnancy weight recalled at the first antenatal visit rather than an objective measurement taken immediately before pregnancy, thus introducing the possibility of recall bias. Moreover, BMI was based on first-trimester weight, which may already include early pregnancy weight gain, leading to potential misclassification—particularly for women near BMI category boundaries. Third, ultrasound accuracy for gestational age assessment may be reduced in women with obesity due to poorer image resolution, potentially leading to non-differential misclassification of preterm birth status. Fourth, inter-pregnancy weight changes were not considered. This is relevant because short IPI in obese women may reflect weight stability and long IPI weight gain, which could bias the optimal IPI estimate. Fifth, our between-women design cannot control for genetic or persistent environmental factors. Hanley (28) et al. used a within-mother matched design and found that the association between short IPI and preterm birth was eliminated in matched analyses (OR 0.85), suggesting that traditional designs may overestimate IPI effects. Therefore, our effect sizes should be interpreted with caution. Sixth, our study lacked data on socioeconomic status, pregnancy intention, and healthcare access—all potential confounders. Reverse causality is also possible: women with prior complicated pregnancies may delay subsequent pregnancies, biasing optimal IPI estimates. These limitations cannot be addressed in our between-women design. Seventh, by excluding IPIs <6 months, we may have introduced truncation bias. This is especially relevant for the obese group, where the estimated optimal IPI lies at the lower boundary (6 months). Moreover, the 95% confidence interval for this estimate is extremely wide (6.0-60.0), indicating low precision. The true optimum could be shorter, or the observed monotonic relationship might be an artifact of this exclusion. However, for normal-weight and underweight women, whose optimal IPIs are well above 6 months, the impact is likely minimal. Therefore, our conclusion for the obese group should be interpreted as “≤6 months (within the range studied)” rather than “exactly 6 months.” Future studies including very short intervals are needed. Eighth, our study did not distinguish between spontaneous and medically indicated preterm birth, which is an important limitation. This is particularly relevant for the obese subgroup, as the proportion of medically indicated preterm births may differ by BMI, and the inability to separate these types could bias the estimated optimal IPI. Finally, as the data were sourced from a single city, caution should be exercised when generalizing the findings.

## Conclusion

This study suggests that pre-pregnancy BMI may be an important effect modifier in the IPI-PTB relationship, with the IPI associated with the lowest PTB risk shortening as BMI increases. This finding indicates that the IPI associated with the lowest PTB risk varies by maternal pre-pregnancy BMI. While observational data cannot support clinical recommendations, the results highlight that uniform IPI guidelines may not be equally suitable for all women. Of note, for women with obesity, the lowest risk was observed at the shortest interval studied (≥6 months), which may reflect a truncation effect rather than a definitive biological optimum. The wide confidence interval further underscores the uncertainty of this estimate. Future prospective studies are needed to test whether BMI-specific IPI intervals would reduce preterm birth. However, the underlying biological mechanisms, such as pathways involving metabolic recovery, inflammatory states, or intrauterine environment, remain unclear and warrant investigation through mechanistic studies incorporating biomarkers. Additionally, prior research has suggested that combining pre-pregnancy BMI with maternal height may improve the prediction of preterm birth, which aligns with our call for more individualized risk assessment and warrants further investigation in future studies (29). Furthermore, our conclusions require validation in prospective cohorts encompassing diverse populations and with precise data on pre-pregnancy and inter-pregnancy weight changes. Future research could also explore whether this interaction exhibits similar patterns for other adverse pregnancy outcomes.

## Data Availability

The datasets presented in this article are not readily available because in accordance with China’s “Interim Measures for Scientific Data Management” (2018), the original datasets used in this study are classified and subject to institutional data governance. To ensure patient confidentiality, raw data are not publicly accessible. Nonetheless, anonymized data that support the findings may be made available to qualified researchers upon reasonable request, contingent upon execution of a data use agreement and approval from both the corresponding author and the overseeing ethics committee. Requests to access the datasets should be directed to Wei-Chao He, heweichao2025@126.com.

## Supplementary material

Supplementary Material 1 Flowchart of Study Population Selection. This flowchart details the selection process of the analytical cohort for this study. The initial data were obtained from the Dongguan Maternal and Child Health Information Platform (2014-2024) and divided into two cohorts: Cohort A (2014-2018) served as a historical matching pool solely for calculating the IPI, while Cohort B (2019-2024) constituted the core analysis cohort. Multiparous women were identified through longitudinal matching based on national ID numbers, performed within Cohort B and between Cohort B and Cohort A. Sequential exclusion criteria were then applied: (1) exclusion of individuals with missing or implausible height/weight data; (2) exclusion of individuals with an IPI outside the study range; and (3) exclusion of individuals with extreme BMI values. Ultimately, 127,542 women were included in the final analysis.

Supplementary Material 2 Heatmap of Crude PTB Rates by IPI and Maternal BMI Categories. This heatmap displays the unadjusted PTB rates (percentage) and corresponding sample sizes (n in parentheses) for each combination of IPI group (rows) and pre-pregnancy BMI category (columns). The color intensity represents the magnitude of the preterm rate, with darker shades indicating higher rates. This preliminary visualization suggests that the relationship between IPI and PTB may vary across different BMI strata.

Supplementary Material 3 Association of IPI and Maternal BMI with PTB Risk in the Unadjusted Model. **(A)** Heatmap, **(B)** Marginal effect of BMI, **(C)** Marginal effect of IPI, and **(D)** Optimal IPI by BMI, derived from the unadjusted model containing only IPI and BMI. The consistent inverse relationship between the optimal IPI and maternal BMI in this model supports the robustness of the primary finding.

Supplementary Material 4 Unadjusted PTB Risk by IPI, Stratified by Maternal BMI. The figure presents the results from the unadjusted generalized estimating equations (GEE) model with RCS, which contained only IPI and BMI categories without any covariates. **(A–D)** The curves depict the unadjusted non-linear relationship between IPI and the predicted probability of PTB across four pre-pregnancy BMI categories. Triangle markers indicate the model-derived optimal IPI for each BMI group. The consistent pattern of shortening optimal IPI with increasing BMI in this unadjusted model supports the robustness of the primary finding from the all-adjusted analysis. *In the figures and their legends, “optimal IPI” is used as a shorthand for “IPI associated with the lowest PTB risk”*.
