# Supplementary figures and images for: Pre-pregnancy BMI modifies the optimal interpregnancy interval for preventing preterm birth: a population-based retrospective cohort study
Source: Front Endocrinol (Lausanne). 2026 Jun 12;17:1762209. doi: 10.3389/fendo.2026.1762209 (PMC13303131; doi:10.3389/fendo.2026.1762209)

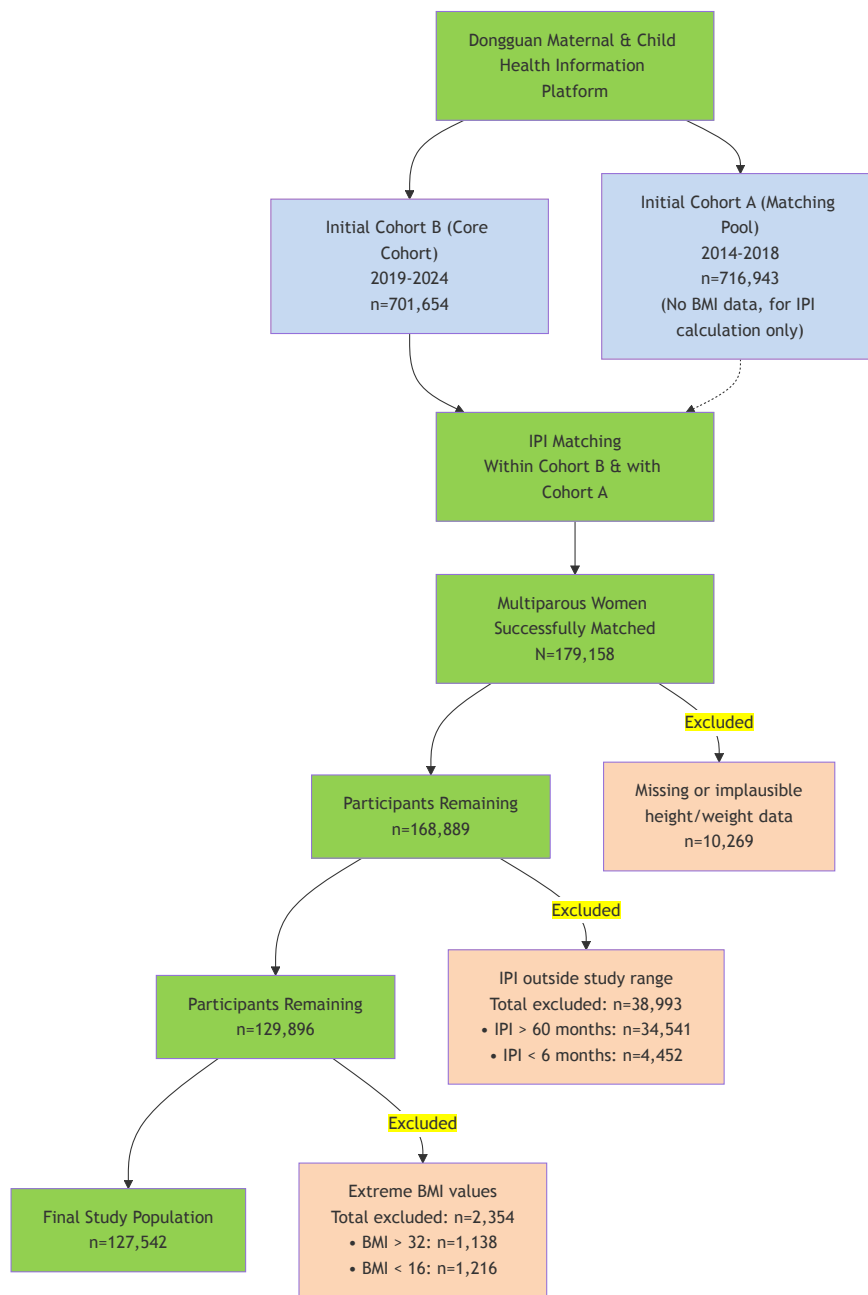

Supplement: Supplementary Material 1 — Flowchart of Study Population Selection. This flowchart details the selection process of the analytical cohort for this study. The initial data were obtained from the Dongguan Maternal and Child Health Information Platform (2014-2024) and divided into two cohorts: Cohort A (2014-2018) served as a historical matching pool solely for calculating the IPI, while Cohort B (2019-2024) constituted the core analysis cohort. Multiparous women were identified through longitudinal matching based on national ID numbers, performed within Cohort B and between Cohort B and Cohort A. Sequential exclusion criteria were then applied: (1) exclusion of individuals with missing or implausible height/weight data; (2) exclusion of individuals with an IPI outside the study range; and (3) exclusion of individuals with extreme BMI values. Ultimately, 127,542 women were included in the final analysis. [file DataSheet1.pdf]
