# Supplementary material for: Pre-pregnancy BMI modifies the optimal interpregnancy interval for preventing preterm birth: a population-based retrospective cohort study
Source: Front Endocrinol (Lausanne). 2026 Jun 12;17:1762209. doi: 10.3389/fendo.2026.1762209 (PMC13303131; doi:10.3389/fendo.2026.1762209)
Supplement: Supplementary Material 2 — Heatmap of Crude PTB Rates by IPI and Maternal BMI Categories. This heatmap displays the unadjusted PTB rates (percentage) and corresponding sample sizes (n in parentheses) for each combination of IPI group (rows) and pre-pregnancy BMI category (columns). The color intensity represents the magnitude of the preterm rate, with darker shades indicating higher rates. This preliminary visualization suggests that the relationship between IPI and PTB may vary across different BMI strata. [file DataSheet2.pdf]

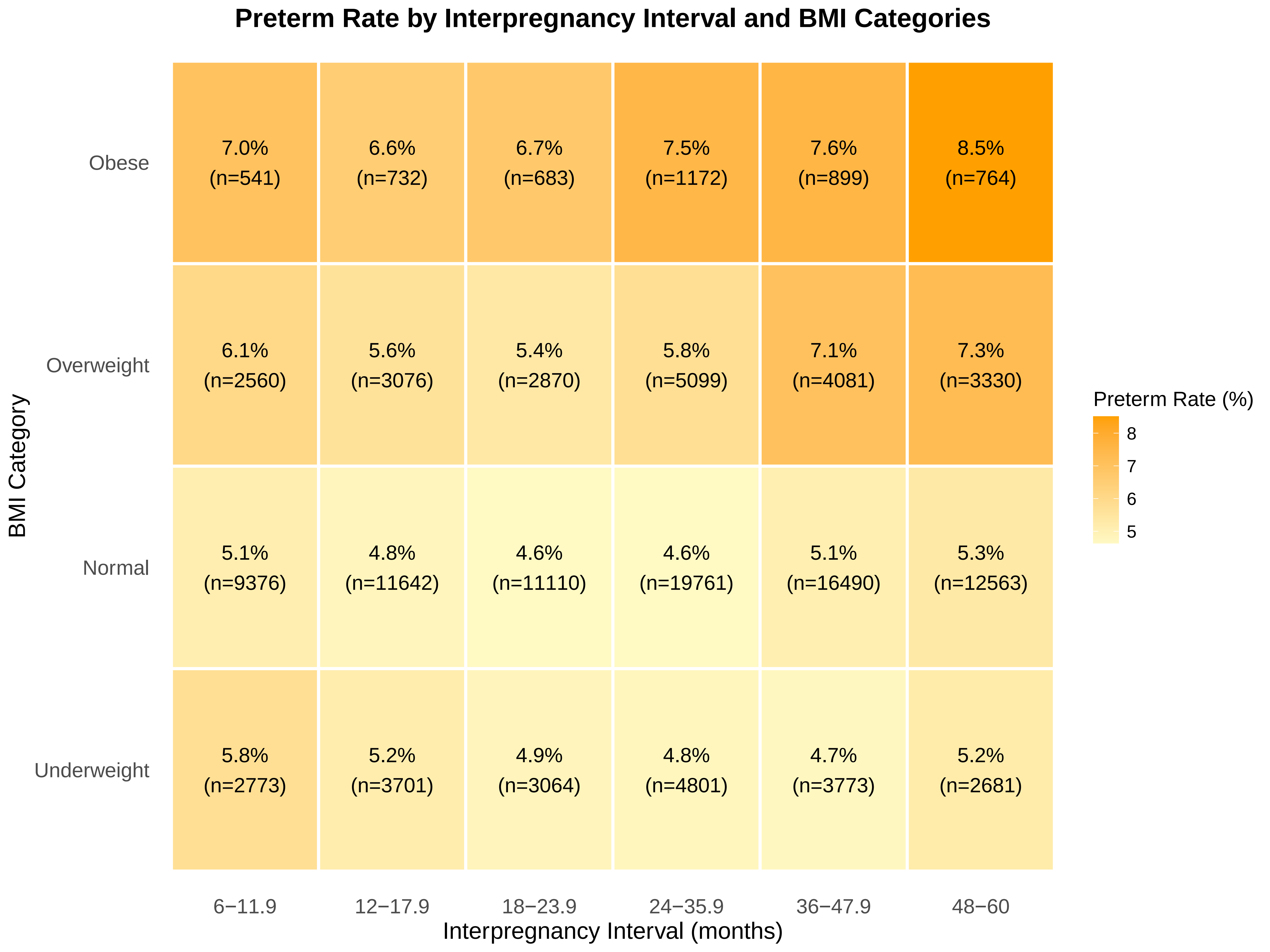
Preterm Rate by Interpregnancy Interval and BMI Categories
BMI Category
Obese
Overweight
Normal
Underweight
7.0% (n=541)
6.6% (n=732)
6.7% (n=683)
7.5% (n=1172)
7.6% (n=899)
8.5% (n=764)
6.1% (n=2560)
5.6% (n=3076)
5.4% (n=2870)
5.8% (n=5099)
7.1% (n=4081)
7.3% (n=3330)
5.1% (n=9376)
4.8% (n=11642)
4.6% (n=11110)
4.6% (n=19761)
5.1% (n=16490)
5.3% (n=12563)
5.8% (n=2773)
5.2% (n=3701)
4.9% (n=3064)
4.8% (n=4801)
4.7% (n=3773)
5.2% (n=2681)
6−11.9
12−17.9
18−23.9
24−35.9
36−47.9
48−60
Interpregnancy Interval (months)
Preterm Rate (%)
8
7
6
5
