# Supplementary material for: Pre-pregnancy BMI modifies the optimal interpregnancy interval for preventing preterm birth: a population-based retrospective cohort study
Source: Front Endocrinol (Lausanne). 2026 Jun 12;17:1762209. doi: 10.3389/fendo.2026.1762209 (PMC13303131; doi:10.3389/fendo.2026.1762209)
Supplement: Supplementary Material 3 — Association of IPI and Maternal BMI with PTB Risk in the Unadjusted Model. (A) Heatmap, (B) Marginal effect of BMI, (C) Marginal effect of IPI, and (D) Optimal IPI by BMI, derived from the unadjusted model containing only IPI and BMI. The consistent inverse relationship between the optimal IPI and maternal BMI in this model supports the robustness of the primary finding. [file DataSheet3.pdf]

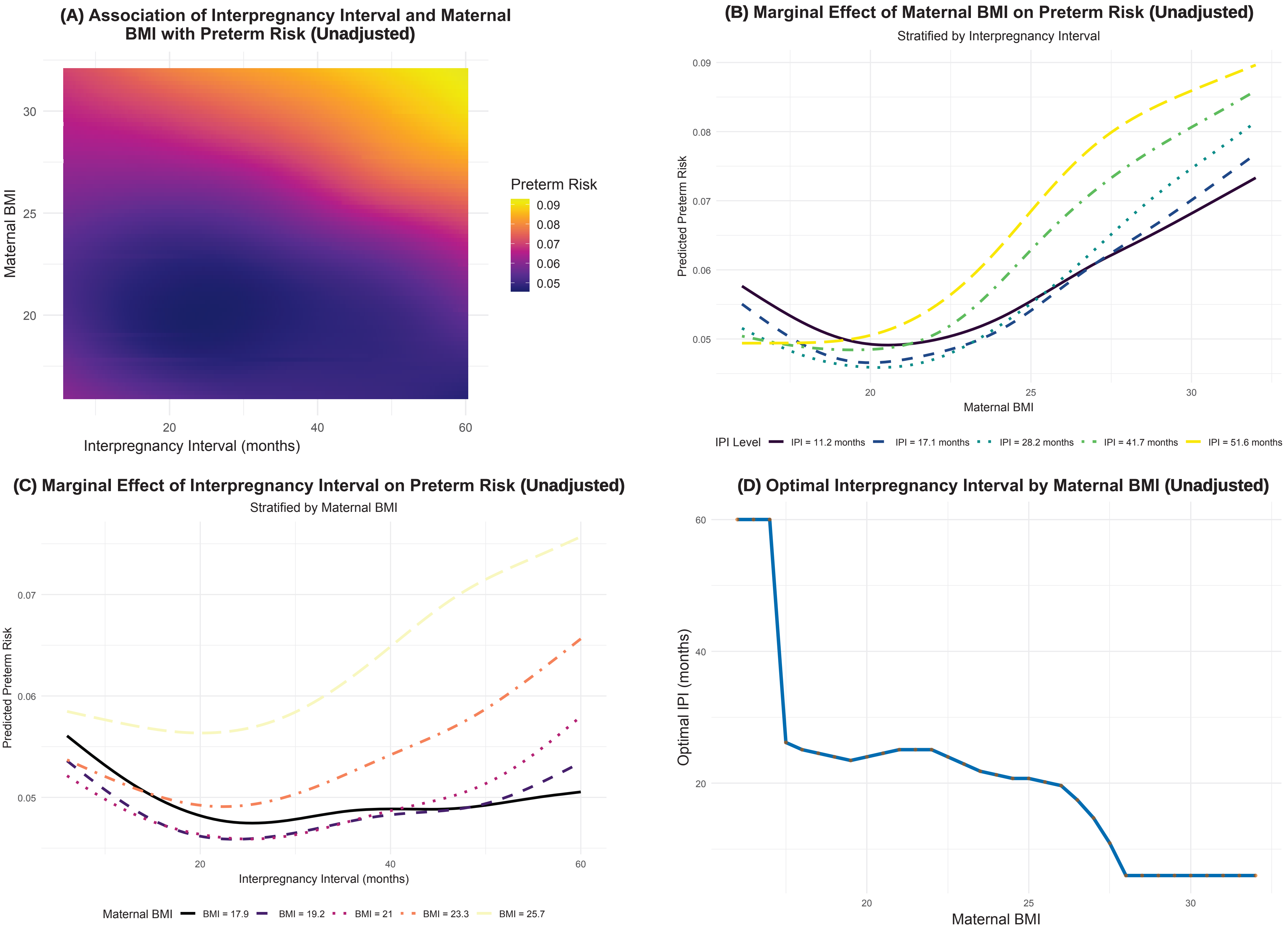
(A) Association of Interpregnancy Interval and Maternal BMI with Preterm Risk (Unadjusted)
Maternal BMI
30
25
20
20
40
60
Interpregnancy Interval (months)
Preterm Risk
0.09
0.08
0.07
0.06
0.05
(B) Marginal Effect of Maternal BMI on Preterm Risk (Unadjusted)
Stratified by Interpregnancy Interval
Predicted Preterm Risk
0.09
0.08
0.07
0.06
0.05
20
25
30
Maternal BMI
IPI Level
IPI = 11.2 months
IPI = 17.1 months
IPI = 28.2 months
IPI = 41.7 months
IPI = 51.6 months
(C) Marginal Effect of Interpregnancy Interval on Preterm Risk (Unadjusted)
Stratified by Maternal BMI
Predicted Preterm Risk
0.07
0.06
0.05
20
40
60
Interpregnancy Interval (months)
Maternal BMI
BMI = 17.9
BMI = 19.2
BMI = 21
BMI = 23.3
BMI = 25.7
(D) Optimal Interpregnancy Interval by Maternal BMI (Unadjusted)
Optimal IPI (months)
60
40
20
20
25
30
Maternal BMI
