# Supplementary material for: Pre-pregnancy BMI modifies the optimal interpregnancy interval for preventing preterm birth: a population-based retrospective cohort study
Source: Front Endocrinol (Lausanne). 2026 Jun 12;17:1762209. doi: 10.3389/fendo.2026.1762209 (PMC13303131; doi:10.3389/fendo.2026.1762209)
Supplement: Supplementary Material 4 — Unadjusted PTB Risk by IPI, Stratified by Maternal BMI. The figure presents the results from the unadjusted generalized estimating equations (GEE) model with RCS, which contained only IPI and BMI categories without any covariates. (A–D) The curves depict the unadjusted non-linear relationship between IPI and the predicted probability of PTB across four pre-pregnancy BMI categories. Triangle markers indicate the model-derived optimal IPI for each BMI group. The consistent pattern of shortening optimal IPI with increasing BMI in this unadjusted model supports the robustness of the primary finding from the all-adjusted analysis. In the figures and their legends, “optimal IPI” is used as a shorthand for “IPI associated with the lowest PTB risk”. [file DataSheet4.pdf]

Unadjusted Preterm Risk by Interpregnancy Interval Across BMI Groups

Triangle markers indicate optimal IPI points (No covariates adjusted)

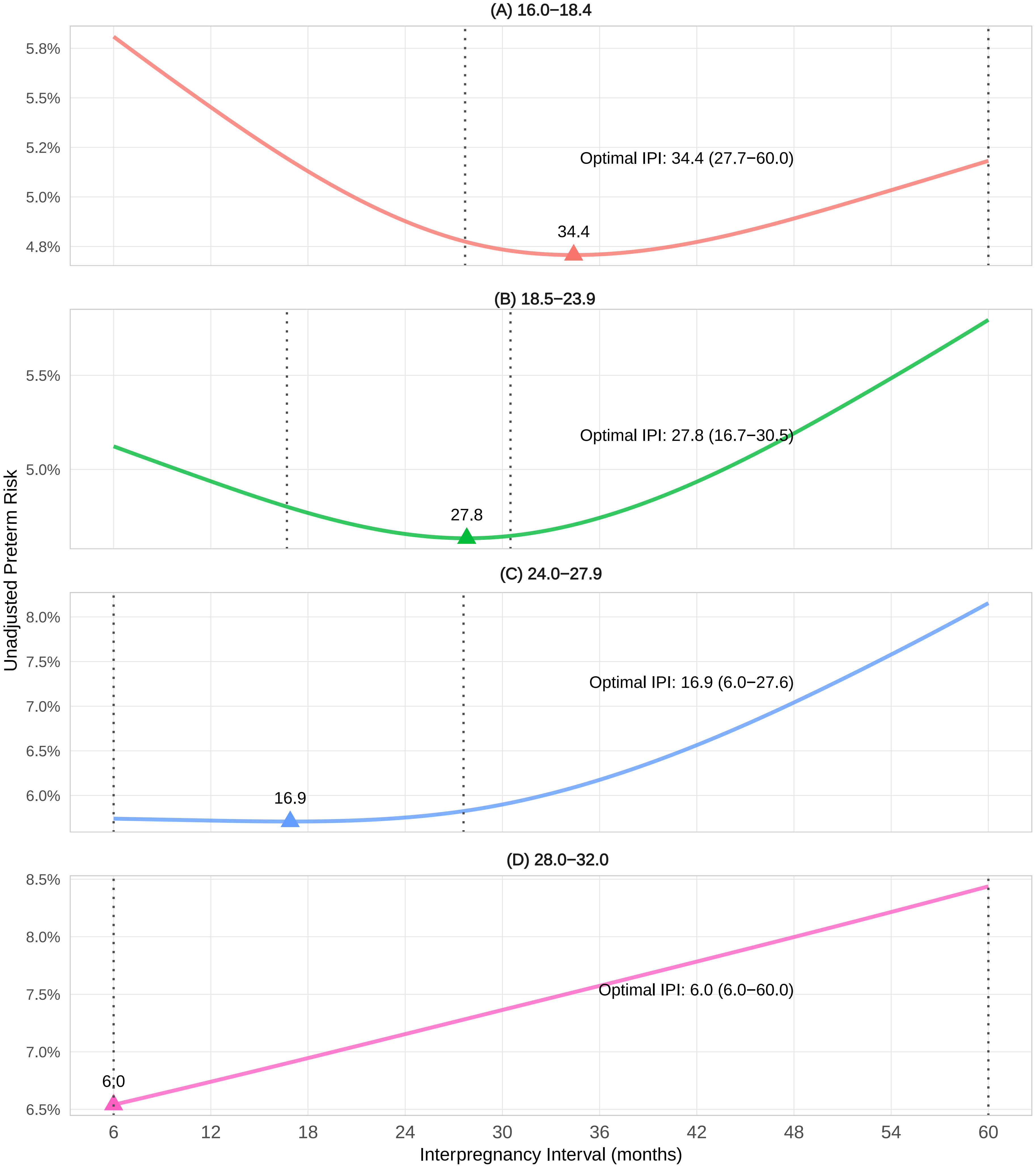

Interaction P−value: Overall P = 0.069 | 18.5−23.9 P = 0.156 | 24−27.9 P = 0.005 | 28−32 P = 0.176
